# Conformational Ensembles by NMR and MD Simulations in Model Heptapeptides with Select Tri-Peptide Motifs

**DOI:** 10.3390/ijms22031364

**Published:** 2021-01-29

**Authors:** V. V. Krishnan, Timothy Bentley, Alina Xiong, Kalyani Maitra

**Affiliations:** 1 Department of Chemistry and Biochemistry, California State University, Fresno, CA 93740, USA; tbentley@csufresno.edu (T.B.); axiong@csufresno.edu (A.X.); 2 Department of Medical Pathology and Laboratory Medicine, University of California School of Medicine, Davis, CA 95616, USA

**Keywords:** NMR, MD, random coil, peptide, ensemble, conformation

## Abstract

Both nuclear magnetic resonance (NMR) and molecular dynamics (MD) simulations are routinely used in understanding the conformational space sampled by peptides in the solution state. To investigate the role of single-residue change in the ensemble of conformations sampled by a set of heptapeptides, AEVXEVG with X = L, F, A, or G, comprehensive NMR, and MD simulations were performed. The rationale for selecting the particular model peptides is based on the high variability in the occurrence of tri-peptide E*L between the transmembrane β-barrel (TMB) than in globular proteins. The ensemble of conformations sampled by E*L was compared between the three sets of ensembles derived from NMR spectroscopy, MD simulations with explicit solvent, and the random coil conformations. In addition to the estimation of global determinants such as the radius of gyration of a large sample of structures, the ensembles were analyzed using principal component analysis (PCA). In general, the results suggest that the -EVL- peptide indeed adopts a conformational preference that is distinctly different not only from a random distribution but also from other peptides studied here. The relatively straightforward approach presented herein could help understand the conformational preferences of small peptides in the solution state.

## 1. Introduction

The direct relationship of the protein’s structure to its function plays an essential evolutionary role in deciding the choice of certain unique combinations of amino acids creating the primary structure leading to a specific three-dimensional conformation. There have been significant advancements in many knowledge-based prediction methods of three-dimensional structures of proteins, including the de novo design of functional manifolds from structural principles [1]. Peptides are dynamic and are known to be important in many functional applications in biomedical sciences [2]. Though peptides can be designed to adopt a specific three-dimensional structure, single mutations in the peptide sequences may influence larger conformational changes. To understand such changes, in this manuscript, we explore the conformational preferences of a set of model heptapeptides using a combination of nuclear magnetic resonance (NMR) spectroscopy and molecular dynamics (MD) simulations. The resultant ensembles were compared with reference to an ensemble of structures if the peptides were to assume random coil conformations.

**Figure 1:**
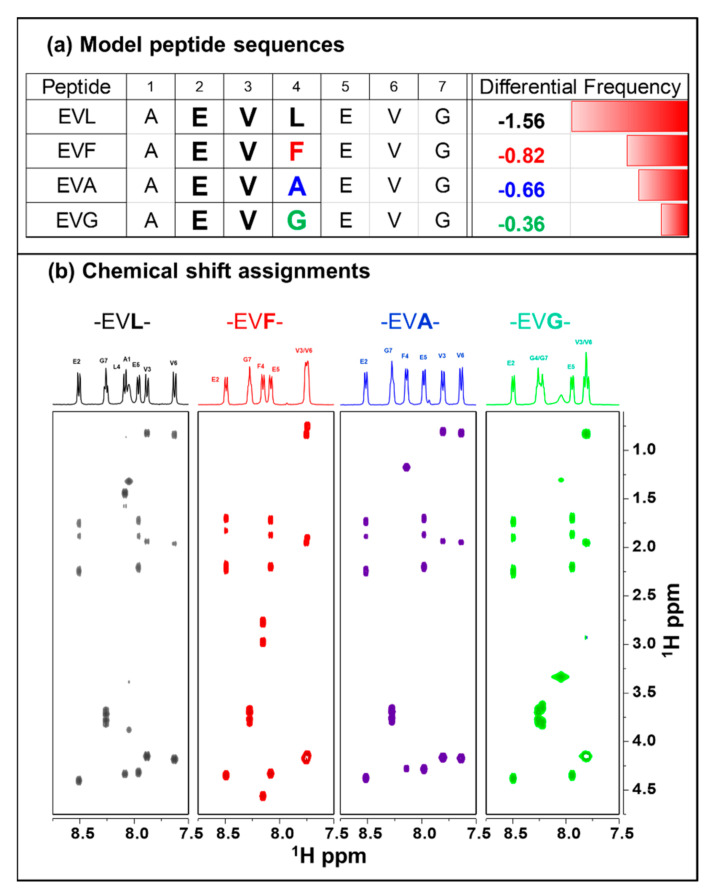
(**a**) List of the model peptides investigated. Heptapeptide sequences a central motif -EVX- with X = L, F, A, or G. For each of the sequences, the corresponding relative frequency of occurrences between globular and transmembrane β-proteins are given. (**b**) Panels of TOCSY spectra of the model -EVX- peptides from left to right: AEVLEVG (black), AEVFEVG (red), AEVAEVG (blue), and AEVGEVG (green). The one-dimensional spectra on the top of each spectrum identify the corresponding chemical shift assignments.

Transmembrane β-barrel (TMB) structures are constituted on the outer cellular membranes of chloroplasts, Gram-negative bacteria, and mitochondria, with a multitude of cellular functions [3]. A peptide motif is defined as a short peptide sequence of a specific pair of amino acid residues separated by a set number of any other amino acids. In this case, a tri-peptide motif is defined by the specific amino acid pair E (aspartic acid) and L (leucine) separated by any of the twenty amino acids defined by *. Gromer et al. have observed this specific preference for certain peptide motifs between the globular proteins and proteins that adopt transmembrane β-barrel structures [4,5]. The peptide motif E*L (* any of the 20 amino acids) appears 1.58 times less in a TMB than in a globular protein—the largest difference found when compared to 400 such tri-peptide possibilities. Such an occurrence in the protein universe must have an evolutionary advantage, particularly when TMB proteins deselect a specific set of tri-peptide motifs. We have chosen an amino acid valine (V) as the central residue in the experimental design, although, in principle, it could be any of the 20 amino acids. The choice of valine is conservative as it would be one of few residues that are not charged, highly flexible or highly rigid, non-aromatic, and do not induce intermolecular bonding. To increase the chances of forming a differentiable structural ensemble between the tri-peptide sequences, the first two residues of the tripeptide are repeated at the C-terminus, leading to a pentapeptide sequence of -EVLEV-. The sequence was amended with A (alanine) and G (glycine) at the N- and C-termini, respectively, to avoid any degeneracy, as a final step, leading to the final heptapeptide sequence, -AEVLEVG-, shown in Figure 1a.

When the C-termini residue of the selected tripeptide is changed to other amino acid residue, the relative frequency of finding such a motif between the TMB and globular proteins also changes. Without duplicating either E or V, there are 18 other possible changes to the L. Residues that would tend to form additional disulfide or hydrogen bonding or charged are avoided leading to three different conservative choices: A representative set of peptides with the following mutations were considered: L > F (phenylalanine), >A (alanine), or >G (glycine). In the -EVX- series of peptides, when X is replaced with F, A, or G, the frequency in TMB proteins reduces by 0.82, 0.66, and 0.36 times, respectively. Figure 1 shows all the representative choice of the amino acid sequence and the differential frequency of occurrences between the TMB and globular proteins.

Ensembles of conformations for all the four peptides are sampled experimentally using solution NMR methods and computationally using the MD simulations. NMR experiments were performed using two-dimensional NMR experiments, and the results are compared with the MD simulation performed over 200 ns. Both the experiments and simulations are performed in dimethyl-sulfoxide. The cumulative investigation of the conformational analyses between the experiments, molecular simulation, and the random coil ensembles suggests that the -EVL- peptide has a preferential adaptation of structures distinctly different from the other peptides. Though the exact reasons are not self-evident from the ensemble of structures, the evolutionary selection of decreased frequency of occurrence of these motifs in TMB may be valid.

## 2. Results and Discussion

### 2.1. Chemical Shift Assignments

Chemical shift assignments of the peptides were done using TOCSY and ROESY experiments. Figure 1b also shows the portions of each peptide’s TOCSY spectra along with the respective identification of amide resonances at the top. A representative example of the sequence-specific assignment in the case of -EVL- peptide is given in Figure S1. The sequence complexity is minimal, leading to a straightforward identification of all the resonances except alanine’s amide proton in some cases. The chemical shifts of both E2 and E5 are well-resolved in all the peptides. The amide regions of -EVL- and -EVA- are much better resolved than the -EVF- (V3 and V6 overlap) and -EVG- (G4 and G7 overlap, and V3 and V6 overlap) at 30 °C. The chemical shift changes in the spectra due to the central residues’ change are not highly significant and range from 0.1 to 0.3 ppm.

### 2.2. Experimental Ensemble of Structures

**Figure 2:**
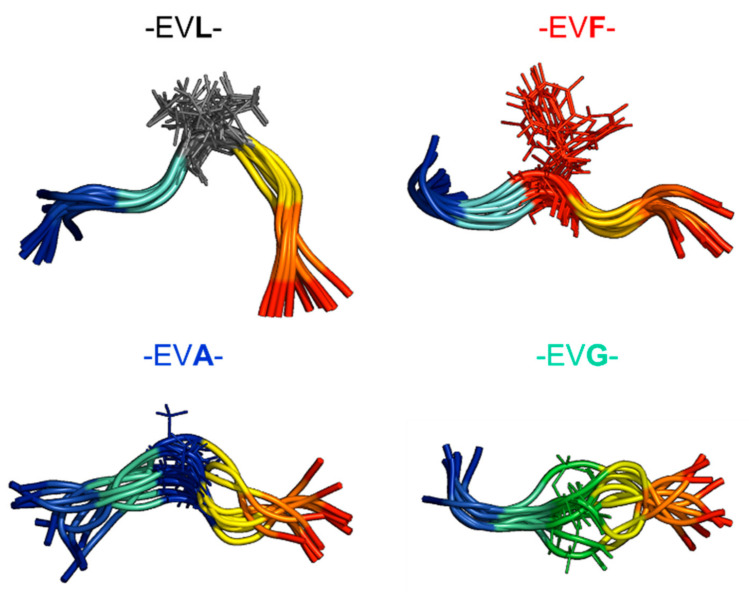
Representative NMR determined structures. An ensemble of the ten lowest energy structures of the peptides was determined using NMR methods. The respective tri-peptide motifs are shown on the top of each ensemble, and the central residues are shown in stick representation with a different color.

The distance restraints derived from the ROESY spectra are used to model the ensemble of structures. Figure 2 shows the representative ensemble of structures (10 lowest energy conformations) of each peptide. The central residue is represented in a stick model in a different color to highlight the location in each case. The calculated backbone RMSD (mean ± std) for the ten structures for each peptide are: -EVL- (1.16 ± 0.39 Å), -EVF- (1.38 ± 0.53 Å), -EVA- (1.90 ± 0.57 Å), and -EVG- (1.93 ± 0.59 Å). As the peptides do not adopt a specific secondary structure conformation, visually, all the ensembles look similar except the -EVL- shows a slight bend around the central residue. Typically, the number of inter-residue constraints in all the peptides are approximately the same (1–2/residue). Considering the molecular weight of the peptide and the spectrometer frequency of 400 MHz, no discernible NOESY peaks were observed (data not shown). The ROSEY spectra of these peptides also do not show any long-range connectivity (|i-j| > 2). The representative ensemble of 10 structures of the peptides do not sample the conformational preferentially between them, and therefore, additional methods are used to explore using a much larger sampling of the conformations.

### 2.3. Conformational Sampling by MD Simulations

**Figure 3:**
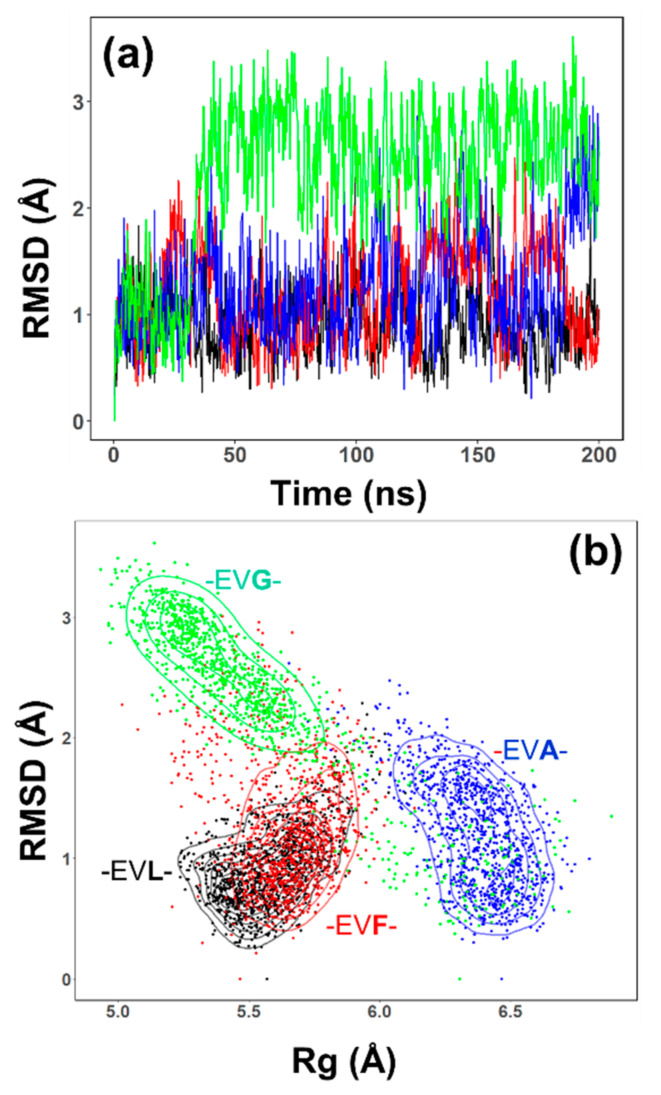
Summary of MD simulations. (**a**) Root-mean-squared deviation (RMSD) of the model peptides as a function of simulation time. (**b**) Correlation plot between the radius of gyration (Rg) and RMSD from the simulation results: -EVX-; X =L (black), =F (red), =A (blue) and G (green).

The representative ensemble generated by NMR methods does show differences between the -EVL-, other peptides; this section focuses on the ensemble of structures sampled by MD simulations. Molecular dynamics simulations (200 ns in explicit solvent) of all the peptides suggest that -EVL- peptide reveals distinct differences in the conformational preferences. Figure 3 highlights the results from the MD simulations of the -EVL- (black), -EVF- (red), -EVA- (blue), and -EVG- (green) peptides. The root-mean-squared deviation (RMSD) values as a function of simulation time for the -EVL- (black) and -EVF- (red) are similar to each other. The RMSD trajectory of -EVG- (green) changes notably after ~ 50 ns to a higher value, while -EVA- (blue) undergoes a similar shift after ~ 150 ns. These trajectory shifts suggest that the central residue (L, F, A, or G) probably influences the conformational dynamics of these peptides.

To further investigate how the overall structural changes are modified, a two-dimensional plot between the RMSD and radius of gyration (Rg) for each of the peptide was done as shown in Figure 3 (-EVL- (black), -EVF- (red), -EVA- (blue), and -EVG- (green)). The two-dimensional plots provide additional resolution to the dynamic features sampled by the peptides. The Rg and the RMSD values of -EVL- and -EVF- have a similar distribution. The -EVA- (blue) peptide shows a conformational sampling of the RMSD similar to -EVL- and -EVF- peptides, but Rg values have shifted to a higher value (from ~5.5 Å to 6.5 Å). In contrast, the -EVG- peptide (green) has a similar Rg distribution to the -EVL, and the -EVF- peptides has an increased RMSD value, as seen in Figure 3a. Also, the -EVG- peptide samples a larger set of values of bother Rg and RMSD showing an elongated distribution than the other peptides, perhaps predominantly influenced by the central glycine residue. The differential dynamics between the peptides are also reflected in the Cα root-mean-square fluctuation (RMSF) (Figure S2). In particular, the -EVG- peptides tend to be much more flexible than the other peptides.

### 2.4. Ensemble of Structures NMR, MD, and the Random Coil Distributions

Inherently the determination of three-dimensional structures by solution-state NMR spectroscopic methods produces an ensemble of structures. The ensemble of conformations modeled is the largest of the various subpopulations that exist as measured by the NMR spectral parameters [6,7,8]. As one of the fundamental objectives, this study enquires if the -EVL- peptide that shows a lower preference of occurrence in TMB proteins than the other sequences adopts a distinct ensemble of conformations. As a corollary to this aim, with the ability to generate an ensemble of structures using molecular dynamic simulations well-parametrized force fields for explicit solvent models, each of the experimental model structures can be compared with the corresponding MD generated ensembles as well as with the respective random coil conformations. This with three different methods for conformation generation for the -EVX- peptides; (a) large ensemble of structures by NMR, based on the ensemble of conformations generated; (b) ensemble of MD structures, based on the simulation results; (c) ensemble of random structures (RC).

**Figure 4:**
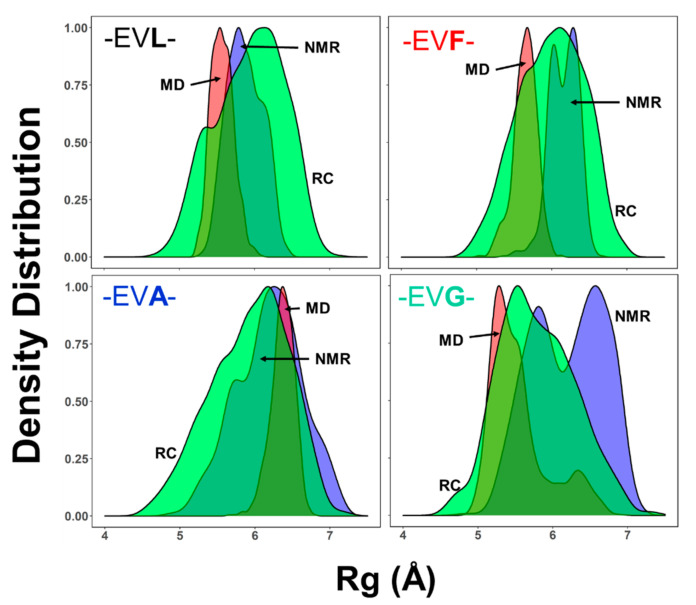
Comparison of the radius of gyration (Rg). Each panel shows the density plots of the distribution of Rg values between the NMR (blue shade), MD (red shade), and random coil (RC, green shade). The data from the peptides are also marked on top of each panel.

One of the commonly used measures to compare the structural features is the radius of gyration (Rg). To investigate the ensemble of structures of these peptide samples, the large ensemble of 1000 conformations are considered. Figure 4 shows the comparison of the ensemble of conformations generated by the three different approaches: an ensemble of random conformations (RC), a large ensemble of NMR structures (NMR), and an ensemble of MD structures (MD). The distribution of the NMR determined ensembles (marked as NMR, blue shade) show some notable variations. The NMR determined Rg distribution of the -EVL- has a mean value of ~5.5 Å and is much smaller than the other peptides (~6–7 Å). While the peptides -EVL-, -EVF- have a narrow distribution of Rg values, both -EVA- and -EVG- have broader distributions, with -EVG- has two distinct populations (see also Figure 3b). The MD determined Rg distributions of the -EVL-, -EVF-, and -EVG- are similar (centered around 5–5.5 Å), while the -EVA- peptide the mean value of shifted to ~6.1 Å. It is also interesting to note that the -EVG- peptide shows an additional set of conformations with a smaller population centered at a slightly higher Rg (~6.1 Å). The random coil ensemble of all the four peptides are broad and relatively similar.

In the case of the -EVL- the Rg value distributions between the NMR and MD simulations are close to each other and mean values are slightly smaller than the distribution sampled by the random coil ensemble (~5.2 Å). This observation suggests that the -EVL- peptide ensemble has more residue-specific conformational distribution than a random distribution. The -EVA- peptide has similar mean values between all the three ensembles, NMR, MD, as well as the random coils at ~6.5 Å. In contrast, both the -EVF- and the -EVG- peptides have lower Rg values by the NMR determined ensembles than the conformations sampled by the MD simulations. In particular, the -EVG- peptide samples a subset of two populations separated ~1.25 Å. This difference in the Rg values for the -EVG- peptide is much larger than the other peptides (-EVF- and -EVA-), showing the relatively larger Rg distribution (Figure 4 and Figure S3). Comparing the Rg distributions of the four peptides within a single conformational sampling method—NMR, MD, or random coil (RC)—is shown in supporting information (Figure S3). In general, the comparison of the Rg distributions between the three methods of sampling (NMR, MD, or RC) among the four different peptides suggests that -EVL- perhaps adopts a slightly more compact conformational distribution than the other three peptides, with -EVG- being the most flexible of the four.

**Figure 5:**
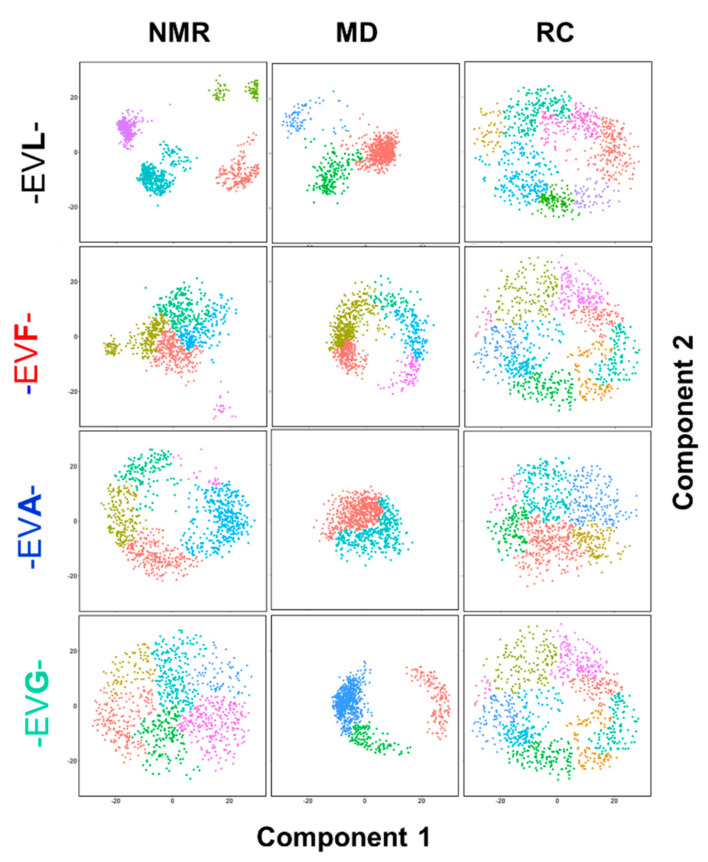
Principal component analysis (PCA) of the ensembles. The plots show the comparison between the PCA of the ensemble of structures (1000 structures each). Each row corresponds to a peptide: -EVL- (top row), -EVF- (second from top row), -EVA- (second from bottom row), and -EVG- (bottom row), the ensemble of conformations estimated by NMR (left column), MD (central column) and random coil (RC, right column) are shown. Components 1 and 2 are plotted across the same scale in both dimensions (−20% to 20%).

Though Rg is an often-utilized measure of structural variations, a detailed analysis of all the structural features using the principal component analysis (PCA) shows a significant difference between the -EVL- and other peptides. The random coil structures of all the peptides, as expected, do not have any distinct clusters (Figure 5, right panels—RC). The NMR determined ensemble of structures of -EVL- shows three tightly formed clusters along with two other minor clusters (Figure 5). The corresponding MD structures of the -EVL- peptide also shows a distinct set of clusters. When taken together, for the -EVL- peptide, both the NMR and MD sample distinct conformations that are notably different from the random coil distributions. The -EVF- peptide, though it does not form distinct clusters as -EVL-, both NMR and MD distributions are different from RC distributions (Figure 5, second panel from top). The PCA of the NMR structures of both the -EVA- and -EVG- peptides suggests that these ensembles of structures are similar to the random coil ensembles. The PCA of the MD structures of these two peptide samples an ensemble that is different from either the NMR or random coil but does not have a distinct separation of subgroups.

Overall, the comparison of the ensemble of structures sampled by NMR, MD, or random coil approaches suggests that the experimentally -EVL- does indeed adopt an ensemble closer to the MD ensemble but distinctly different from the random coil ensemble. For the -EVF- peptide, the distinction between the NMR and MD ensembles concerning the random coil structures is less pronounced than the -EVL peptide. However, both the other two peptides (-EVA- and -EVG-) have the experimental ensembles close to the random coil ensembles.

## 3. Materials and Methods

### 3.1. Peptide Synthesis

The peptides were synthesized in-house by the standard solid-phase synthesis (SPS) protocol using the microwave-assisted peptide synthesizer Liberty Blue (CEM Corporation, Matthews, North Carolina, USA). The resins, amino acids, and Oxyma pure were obtained from the same vendor. Dimethylformamide (DMF), diisopropyl carbodiimide (DIC), 4-methyl piperidine, acetonitrile, and all other chemicals were purchased (Sigma-Aldrich, St. Louis, MO, USA). The synthesized 7-mers were cleaved from the resin using a cocktail containing 92.5% trifluoroacetic acid (TFA), 2.5% deionized water, 2.5% triisopropylsilane (TIS), and 2.5% dioxa-1,8-octane-dithiol (DODT). The cleaved peptides were precipitated by the addition of ether and then redissolved in a 1% acetonitrile-water mixture. The solutions were lyophilized to obtain a solid powder of the peptides with a percentage yield ranging between 34–66%. The purity for the peptides was checked by high-performance liquid chromatography (HPLC) on a Shimadzu instrument using a Luna 5 μm C18, 100 Å, 250 × 4.6 mm reverse-phase analytical LC column with a gradient of 5% to 70% acetonitrile in water (both containing 0.1% (*v*/*v*) trifluoroacetic acid). All the NMR sample tubes were vacuum-sealed to avoid the absorption of water molecules by the solvent with a ~26 mM concentration in deuterated dimethyl sulfoxide.

### 3.2. NMR Spectroscopy

The experiments for determining the peptides’ three-dimensional conformation using NMR spectroscopy followed a previously established standard procedure [6,7,8]. All the NMR experiments were performed on a Varian-Agilent 400 MHz NMR spectrometer. A double resonance NMR probe (one-NMR probe) with a z-axis pulsed field gradient (PFG) was used. In addition to standard one-dimensional ^1^H experiments, two-dimensional ^1^H-^1^H experiments were performed. A ^1^H-^1^H TOCSY experiment with an isotropic mixing (80 ms) with a DIPSI [9] sequence (B_1_ field strength 7.5 kHz) was used for chemical shift assignment. Because of the molecular size and spectrometer field strength, only rotating frame nuclear Overhauser effect (ROE) experiments effectively led to the collection of ^1^H-^1^H ROESY [10,11] experiments for all the peptides. ROESY experiments were performed with an adiabatic spin-lock field of 4.0 kHz. All the two-dimensional experiments were performed in phase-sensitive mode with 2048 points in t_2_ and 256 points in the t_1_ domains and with 64 transients for each increment. A relaxation delay of 2.0 s was used between the scans. The sensitivity of the NMR spectra across the samples were comparable. Three-bond ^1^H-^1^H coupling constants were measured from one-dimensional spectra. All the experiments were performed at 30 °C unless otherwise mentioned. NMR data were processed using a combination of NMRPipe [12] and Sparky [13,14], while the spectral figures were made using Mestrenova^®^ (http://mestrelab.com/).

### 3.3. Ensemble of NMR Structures

The conversion of the NMR experimental parameters to three-dimensional conformation was also performed using standard processes [6,7,8]. The distance constraints were derived from the ROESY cross-peak volumes. Additional dihedral angle constraints were derived using the three-bond (^3^JHNα) coupling constants using the Karplus equation and backbone chemical shifts using TALOS [15,16]. The experimental constraints were then used within the framework of CYANA to generate the three-dimensional structural models [17,18]. Starting from 50,000 random conformations, two sets of ensembles of structures were generated. (a) The large ensemble of NMR structures: a set of 1000 structures and (b) a representative ensemble of NMR structures, a subset of 10 lowest energy structures from the set (a). The large ensemble was used for comparison with the other methods (see below). The CYANA generated structures had no constraint or van der Waals violations.

### 3.4. Generation of Random Structures

A set of random structures for all the four pepti des were generated using TraDES-2 [19]. A random selection (without repeat) of 1000 structures were chosen and referred to as an ensemble of random structures.

### 3.5. Molecular Dynamics Simulations

The molecular dynamics simulations were performed using the Desmond [20] using the academic implementation interface Maestro by Schrödinger [21]. Starting from the primary sequence (FASTA format), the initial atomic coordinates were generated in an extended conformation for each peptide using PyMol [22]. The peptides’ three-dimensional coordinates were imported into Mastero, optimized, and refined using the ‘Protein Preparation’ tool. Each peptide system was built with an explicit solvent model of DMSO within an orthorhombic box. A default relaxation process for the isothermal-isobaric ensemble (NPT) ensemble was adopted following the six steps described in the manual [21]. The temperature (299 K) and pressure (1.013 bar) were set by the Nose-Hoover thermostat and Martyna-Tobias-Klein methods. Each peptide was simulated for 200 ns with the default simulation parameters, NPT conditions, 299 K, 1.013 bar, OPLS-AA 2005 force field, and SHAKE algorithm with two fs each for bonded and near interactions and six fs for far interactions. Initial validation of the MD results was performed using the ‘simulation analysis’ tools within Desmond. Additional detailed analyses were performed using the Bio3D developed by Grant et al. [23,24].

A combined analysis of the ensemble of structures was performed using in-house codes written in R-statistical programming [25].

## 4. Conclusions

A theoretical calculation of primary structures suggested that certain dipeptide motifs have relatively less occurrence in transmembrane β-barrel proteins than in globular proteins. This study investigated the ensemble of conformations sampled by incorporating one such tri-peptide motif -EVL- in the N-termini part of the heptapeptide using NMR, MD, to explore if the peptide were to assume a random coil distribution. The ensemble of structure sampled by -EVL- is compared with three other peptide sequences where the L- replaced by F, A, or G. Though none of the peptides adopt a well-defined secondary structure, the study results suggest that -EVL- indeed sample a conformational ensemble that is distinctly different from the other three peptides. The observations are of importance because even in model peptides such as the ones chosen here, the choice of the ensemble of conformations the peptides, albeit small, is influenced by the peptide’s primary structure.

The choice of the model peptide -AEVXEVG-, though presented with a rationale, is one of the multiple possibilities. The tri-peptide sequence -E*L- occurs 1.56 times in globular proteins than in a TMB, while the next tri-peptide motif -E*K- occurs 1.24 times less. By the same comparison, the tri-peptide -S*S occurs about 1.6 times more in a globular protein than in a TMB as phosphorylation of the TMB is an evolutionarily essential functional requirement selected. The exact reason for deselecting an E*L motif in a TMB is not apparent from this study. Nevertheless, the heptapeptide’s conformational ensemble with an -EVL shows an ensemble distinct from random coil conformation and the other three peptides with a single residue change.

In comparing the ensembles, this study samples 1000 structures for the NMR, MD, and random coil distributions. Even when using the (φ, ψ) torsion angles as the degrees of freedom, a protein chain has an infinite number of different conformations. The NMR based structures have the advantage of selecting a subset of these conformations that matches the experimental restraints, while the solvent model within the MD engine is also optimal for conformational selection. Nonetheless, as expected, a closer match between the NMR and MD ensemble may not be achievable considering the flexibility of the short peptides used in this study. Despite these limitations, this study reiterates that estimating the ensemble of a peptide’s structures is more meaningful than presenting a single structure as the ensemble defines a factual nature. In a broader sense, the evolutionary selection of specific primary sequences in a protein may also be influenced by the ensemble of conformations [26,27].

## Data Availability

The data presented in this study are available on request from the corresponding author as we could not identify a public database for depositing such data.

## Supplementary Materials

The following are available online at https://www.mdpi.com/1422-0067/22/3/1364/s1, Figure S1: Representative chemical shift assignments of -EVL- peptide using a ROESY spectrum. Figure S2: Plots for the root mean squared fluctuations (RMSF) of the Cα atoms from the model peptides’ 200 ns MD trajectories. Figure S3: Comparison of the radius of gyration profiles: NMR (top), MD (middle), and RC (Random coil, bottom). The peptides are identified in each plot.
