# Supplementary figures and images for: Conformational Ensembles by NMR and MD Simulations in Model Heptapeptides with Select Tri-Peptide Motifs
Source: Int J Mol Sci. 2021 Jan 29;22(3):1364. doi: 10.3390/ijms22031364 (PMC7866422; doi:10.3390/ijms22031364)

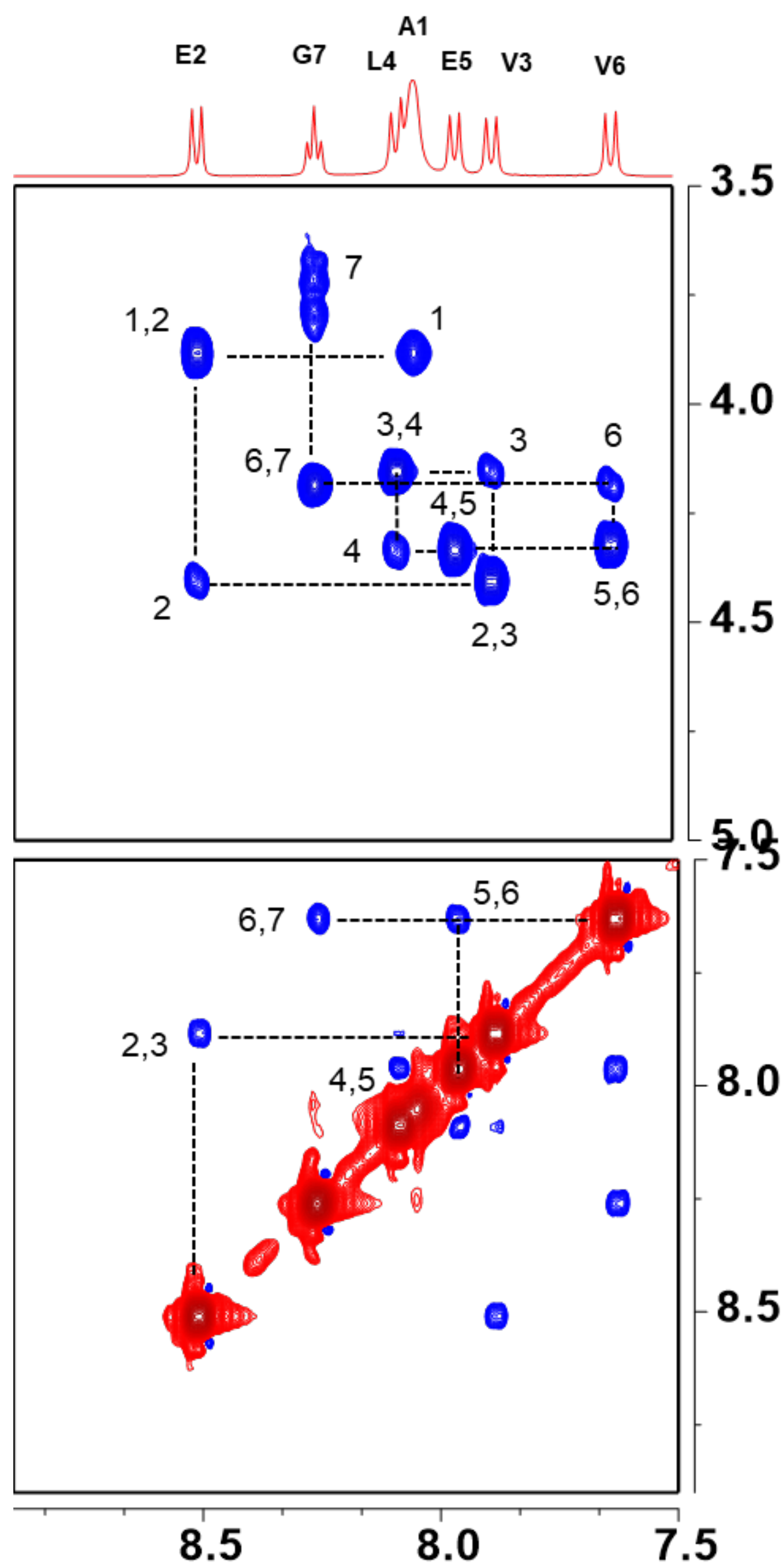

Figure S1

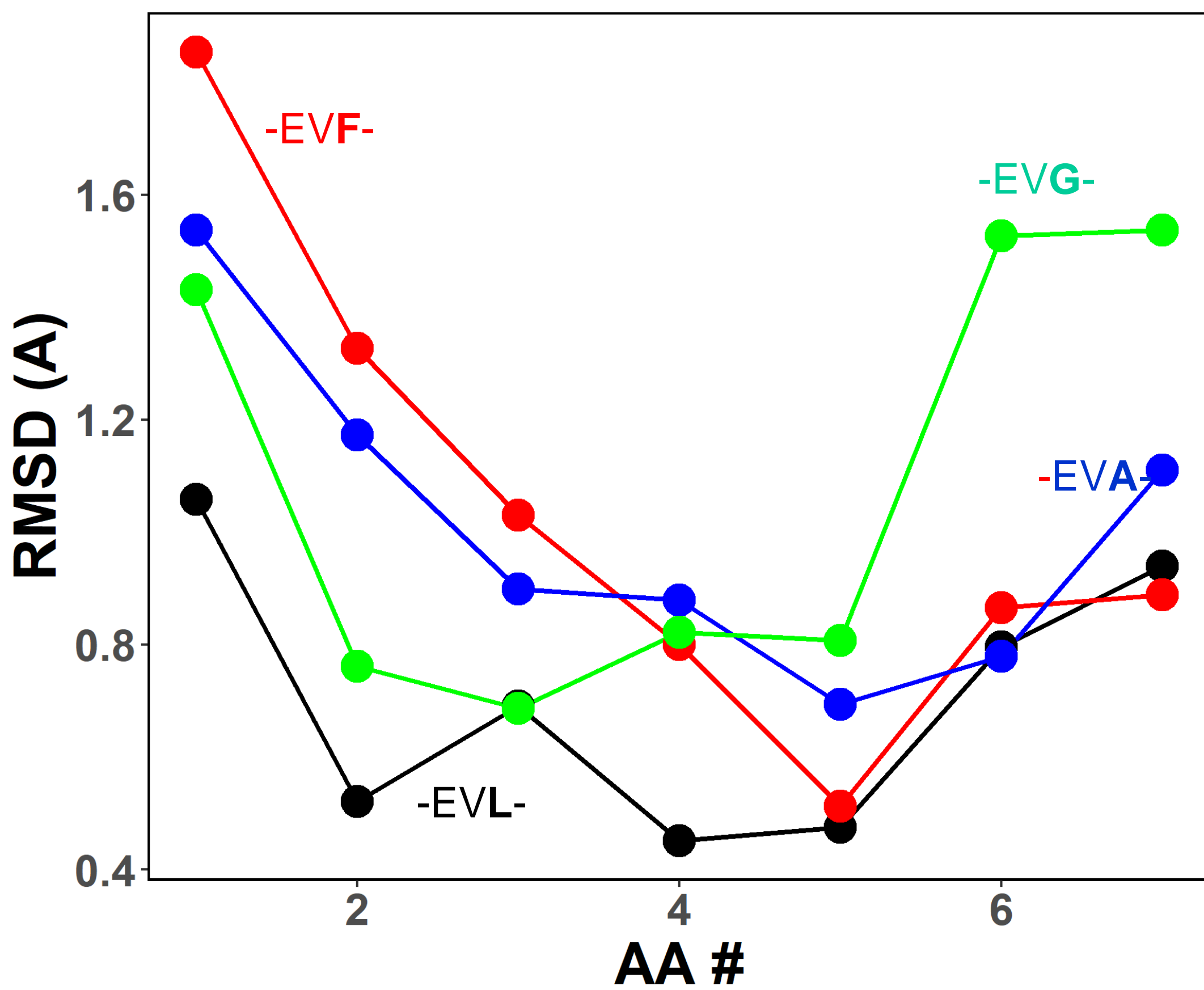

Figure S2

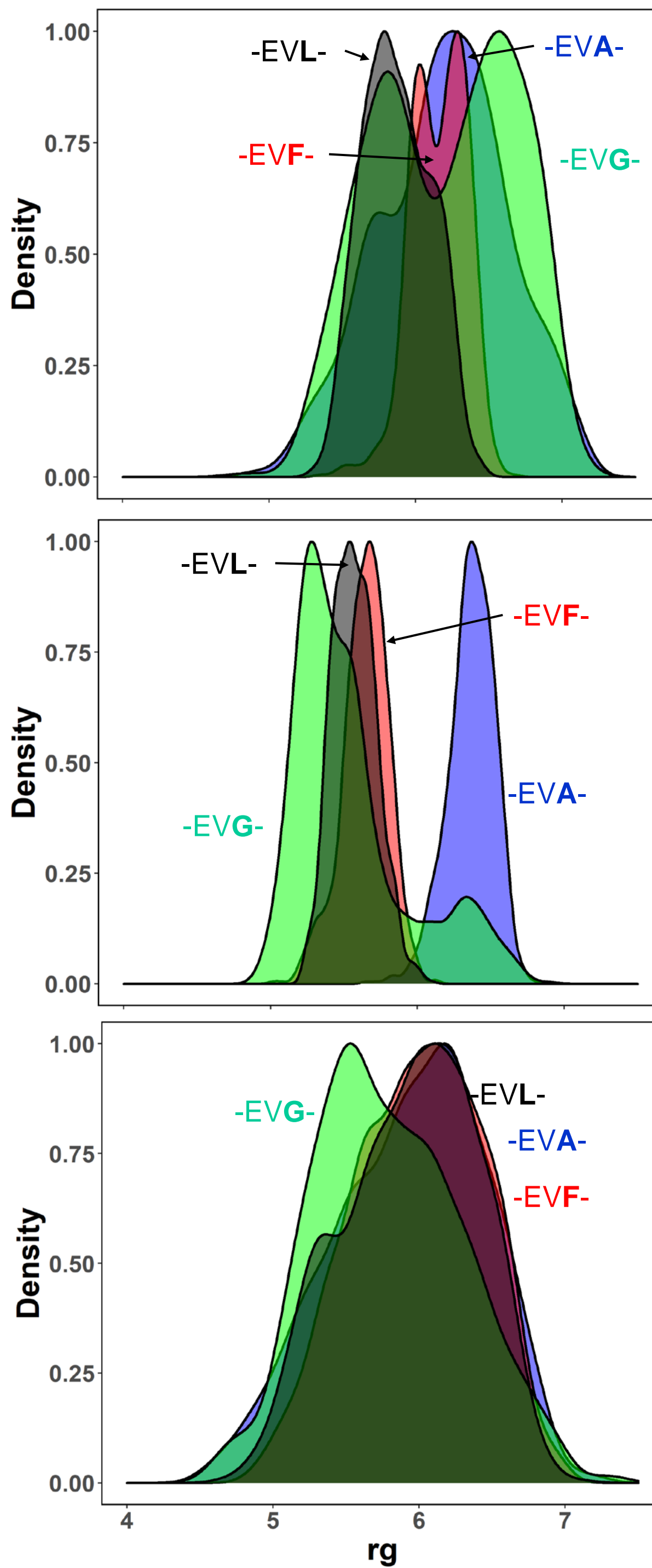

Figure S3

Supplement: Supplementary file 1 [file ijms-22-01364-s001.pdf]
